# The history of robotic surgery and its evolution: when illusion becomes reality

**DOI:** 10.1590/0100-6991e-20202798

**Published:** 2021-01-04

**Authors:** ANDRE LUIZ GIOIA MORRELL, ALEXANDER CHARLES MORRELL-JUNIOR, ALLAN GIOIA MORRELL, JOSE MAURICIO FREITAS MENDES, FRANCISCO TUSTUMI, LUIZ GUSTAVO DE-OLIVEIRA-E-SILVA, ALEXANDER MORRELL

**Affiliations:** 1 - Instituto Morrell, Cirurgia do Aparelho Digestivo Robótica e Minimamente Invasiva - São Paulo - SP - Brasil; 2 - Sociedade Beneficente Israelita Brasileira Albert Einstein, Cirurgia Geral e do Aparelho Digestivo Minimamente Invasiva e Robótica - São Paulo - SP -Brasil; 3 - Rede D’Or São Luiz, Cirurgia do Aparelho Digestivo Robótica e Minimamente Invasiva - São Paulo - SP - Brasil; 4 - Hospital Vila Nova Star, Cirurgia do Aparelho Digestivo Robótica e Minimamente Invasiva - São Paulo - SP - Brasil; 5 - Grupo Leforte, Cirurgia do Aparelho Digestivo, Bariátrica e Metabólica Robótica - São Paulo - SP - Brasil; 6 - Hospital Alemão Oswaldo Cruz, Departamento de Cirurgia - São Paulo - SP - Brasil; 7 - Hospital Federal de Ipanema, Cirurgia Geral - Rio de Janeiro - RJ - Brasil

**Keywords:** Robotics, Robotic Surgical Procedures, Telesurgery, Exoskeleton Device, Virtual Reality, History of Medicine, Tecnology, Robótica, Procedimentos Cirúrgicos Robóticos, Telecirurgia, Exoesqueleto Energizado, Realidade Virtual, História da Medicina, Tecnologia

## Abstract

The term “robot” was concepted in the beginning of last century, coming originally from the Czech word “robota”, meaning “labor”. More recently, computer assistance and robotics based in the telepresence and virtual reality concept have been applied to surgical procedures. The application of robots in surgery dates approximately 35 years, experiencing significant growth in the last two decades fueled by the advent of advanced technologies. Despite its recent and brief status in surgery history, robotic technology has already proven its enhanced visualization, superior dexterity and precision during minimally invasive procedures. Currently, the worldwide diffused and predominant robot system used in surgery is Da Vinci by Intuitive Surgical, however robotic surgery evolution is far from over, with multiple potential competitors on the horizon pushing forward its paradigms. We aim to describe the history and evolution of robotic surgery in the last years as well as present its future perspectives.

## RESUMO

O termo “robô” foi concebido no início do século passado, derivado originalmente da palavra tcheca “robota”, que significa “trabalho”. Mais recentemente, a tecnologia de computação associada à robótica, baseada no conceito de telepresença e realidade virtual, têm sido aplicadas aos procedimentos cirúrgicos. A aplicação de robôs em cirurgia data de aproximadamente 35 anos, experimentando um crescimento significativo nas últimas duas décadas impulsionado pelo advento de novas tecnologias e seus resultados. Apesar de seu status breve comparado à longevidade da história da cirurgia, a tecnologia robótica já provou seus potenciais benefícios com visualização aprimorada, destreza superior e maior precisão durante procedimentos minimamente invasivos. Atualmente, a plataforma robótica mundialmente difundida e predominantemente usada em cirurgia é o modelo Da Vinci da empresa Intuitive Surgical, e a evolução desse novo conceito de cirurgia está longe de terminar, com inúmeros competidores potenciais no horizonte impulsionando a quebra de paradigmas. Nosso objetivo nesta revisão é descrever a história e evolução da cirurgia robótica nos últimos anos, bem como apresentar suas perspectivas futuras.

## INTRODUCTION

Although the term and existence of “robots’’ is relatively new, the idea of autonomously operating machines can be dated for centuries. The term “robot” was concepted by Joseph Capek in 1921, in his play Rossom’s Universal Robots, which originally came from the Czech word “robota”, meaning “labor”. The term rapidly became corrupted to reflect a machine-oriented repetitive task. Computer assistance, robotics, automation, and virtual reality are quite new concepts, and more recently they have been applied to healthcare assistance. The last decades have witnessed an exponential growth in medical technology, with the robotic platform applied to surgery one of its most remarkable events. Robots were used in the surgical world more than 30 years ago, and have become a new standard of care, yielding interesting results. The purpose of this article was to describe the history, evolution, current status, and perspectives of robotic surgery. 

## BACKGROUND

The idea of creating an automatic machine able to carry out some tasks usually performed by human hands is quite old. Regarding its surgical applications, its initial concept began more than 60 years ago in the military field. During military combats, hostile environments whose access is difficult and sometimes undesirable are frequent, therefore, lacking proper healthcare assistance. Not rarely, the area with the most limited resources and manpower is the closest to the site of injuries. Hemorrhagic shock and polytrauma are the most frequent causes of primary death in combat, and therefore, for the military there was an immediate need to provide expert surgical care after major trauma based on damage control surgery^1^. Changing the “Golden-Hour paradigm”, instead of transferring the injured soldier to the closest hospital, the new concept was to bring the operating room to the closest support unit, allowing a faster intervention in a “Golden-minute” approach. Another important scenario contributing to robotics and the telepresence concept development was the space race. In 1957, a Soviet R-7 intercontinental ballistic missile launched Sputnik (Russian for “traveler”), the world’s first artificial satellite and the first man-made object to be placed into the Earth’s orbit. A year later, the United States launched its own satellite and created the National Aeronautics and Space Administration (NASA), a federal agency dedicated to space exploration. Not different from warzone, space is the most obvious scenario where human presence demands huge efforts. The necessity for instrument telemanipulation resulted in private and national public engagement for technology development in the area. The virtual reality pioneer Scott Fisher developed the first head mounted display (HMD), which immersed the viewer in a three-dimensional (3D) virtual environment while the engineer Phil Green developed a system of robotic telemanipulation for microsurgery at the Stanford Research Institute (SRI). Both ideas of telepresence and robotized telemanipulation were essential to assure the concept of telesurgery.

### The Robotic Era

The first robot platform used on a human patient was used in 1985 to perform neurosurgical biopsies, called Programmable Universal Machine for Assembly 200 (PUMA)^2^. Further, the machine was adapted and used for common urologic and prostate procedures by The Robotics Centre at Imperial College^3^. In 1992, an image-guided system named Robodoc® Surgical System was developed for the use in prosthetic hip replacement^4^. Allowing the surgeon to optimize the prosthesis size on a patient-specific basis, the Robodoc is currently the only active robotic system cleared by the Food and Drug Administration (FDA) for the use in orthopedic surgery. 

In the 90s of the last century, driven through scientists’ efforts, a paradigm shift occurred in the robotic scenario adapting the masterslave concept, which represented remote control of robotic movements by a distant workstation. Funded in 1990 by Yulun Wang, endorsed by the Defense Advanced Research Projects Agency (DARPA) and, probably, encouraged by Jacques Périssat’s. presentation, in 1989 at the SAGES annual conference in Atlanta, a company called Computed Motion initially developed a voice-controlled robotic arm equipped with an endoscope named AESOP® (Automated Endoscopic System for Optimal Positioning). 

**Figure 1:**
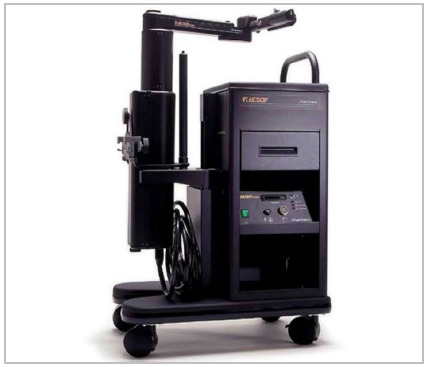
AESOP (Automated Endoscopic System for Optimal Positioning).

The first model of the robotic arm approved in 1994 for usage, the AESOP 1000, was controlled using pedals. Its future generation, the AESOP 2000 designed 2 years later, replaced the pedals with a voice control system, allowing the surgeon to have control of the endoscope, providing a ‘‘third hand” (Figure 1). By using its voice, the AESOP 2000 eliminated the necessity of an assistant to hold the endoscope. The platform evolved to AESOP 3000® increasing the degrees of freedom, and had its final platform with the AESOP HR (HERMES Ready), having integrated voice control and functions such as operating room lighting and movement of the operating table. When idealized, the robotic AESOP was designed to improve image stability and reduce the medical personnel required in the operating room, showing numerous documented advantages over traditional human-assisted camera holding, especially replacing the need for a surgical assistant who may become fatigued during long procedures^5^. Not completely satisfied, however, the surgical procedures demanded not only the concept of telemanipulation of the video camera but also surgeons’ movements. 

**Figure 2:**
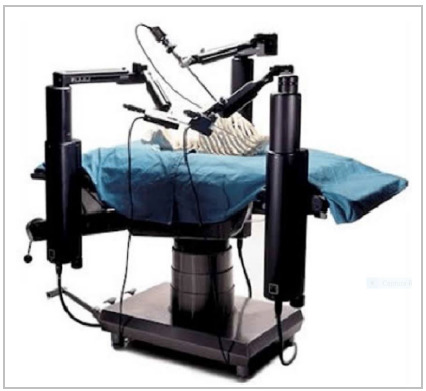
ZEUS robot consisted of three arms.

As a result, Computer Motion, in 1998, presented the Zeus system with arms and surgical instruments controlled by the surgeon, introducing the actual concept of telepresence, in which the surgeon (master) commands the slave (robot). The ZEUS robot consisted of three arms, each independently attached to a surgical table, having one AESOP arm controlling the scope and two other surgical arms with four degrees of freedom (Figure 2). The surgeon console consisted of a video monitor and two handles which are able to manipulate the instruments, providing an enhanced interface and 2-dimensional display. The ZEUS robotic platform was used for the first time in 1998 at the Cleveland Clinic for uterine tube anastomosis surgery^6^. One year later, an open-heart grafting of the coronary artery was performed in Canada. In 2001, a major step in robotic surgery took place in what was called the Operation Lindberg. In allusion to Charles Lindberg, the American aviator who performed the first solo transatlantic flight, the Operation Lindberg consisted of a robotic surgery performed using the ZEUS robot and a telecollaboration system SOCRATES that allowed a surgeon using a remote operating station to control the robotic arm located across the Atlantic. The procedure was a cholecystectomy done by Jacques Marescaux in New York on a patient situated in Strasbourg, France^7^. The procedure lasted 54 minutes and had no technical incidents, and with no perception of distance. 

Years before, in 1995, Frederick H. Moll and Robert Younge founded the Intuitive Surgical in California, reworking the surgical telepresence system developed by Phil Green of SRI and developed the Intuitive’s first robotic surgical prototype: Lenny, an abbreviation of Leonardo. The patient-side cart of Lenny consisted of 3 separate robotic arms that clamped to the operating table, where two were instrument arms and the third arm held the scope camera^8^. The Intuitive’s second generation was Mona, in 1997, being their first robotic surgical system to be used in human trials. Mona’s first procedure was a cholecystectomy performed in 1997 by Jacques Himpens, MD, a bariatric surgeon out of Saint-Blasium General Hospital in Dendermonde, Belgium^9^. Further procedures were achieved such as arteriovenous fistula cases and gastric banding procedures^10^ ^,^ ^11^. In 1998, Intuitive brought into human use the first system of what would be the most successful robotic surgery platform until now: da Vinci. The procedures were diverse, including thoracoscopic internal mammary harvesting, mitral valve repair, cholecystectomies and Nissen fundoplication^8^. The initial commercial sale of the da Vinci was to the Leipzig Heart Center in Germany in late 1998, and with its aid, the surgeons were able to perform a cardiac valve repair and coronary artery bypass graft surgery that were significantly less invasive than the standard sternotomy^12^. In 2000, the da Vinci obtained FDA approval for general laparoscopic procedures and became the first operative surgical robot in the United States (US). 

In 2003, following a 3 year legal battle, the Computer Motion merged with the Intuitive Surgical into a single company, discontinuing the development of the ZEUS system. Both companies combined the efforts having many of its elements integrated with later projects in producing a more effective technology.

### The da Vinci Era

Despite the 20 year existence of the Intuitive Surgical Inc, the current Da Vinci platform represents approximately 35 years of combined efforts and technology in favor of robotic surgery. During its evolution, some key points have been interesting to reinforce its actual hegemony and worldwide dissemination. Encouraged by the telepresence surgery concept created by Phil Green, Richard Satava and the Stanford Research Institute (SRI), the government DARPA project resulted in a prototype robot mounted into an armored vehicle that could ‘‘virtually’’ take the surgeon to the front lines^13^. The vehicle model was the Medical Forward Area Surgical Team (MEDFAST) that could be driven directly to the battlefront while the surgeon console would be protected in a Mobile Advanced Surgical Hospital (MASH), operating at a safe distance from the battlefield. Through this technology, the first remote telesurgical procedure having an intestinal anastomosis on ex-vivo porcine was a landmark to inspire Frederick H. Moll to create Intuitive Surgical and boost the telepresence concept. 

Compared to the ZEUS platform, the Intuitive’s next generation system, da Vinci, improved significantly on the previous prototypes. The robotic system was composed of 3 components, with a patient cart, a surgeon console and the image system. All robotic arms originated from a single patient cart, which obviate the need to mount each arm to the operating table and solved issues with table positioning. With seven degrees of freedom and two degrees of axial rotation, the surgical instruments imitate the human wrist. The surgeons console with an image system brought to da Vinci robot a completely innovative manner to connect the surgeon with the stereoscopic viewer, having a binocular visualization trademark. Instead of a video display, the viewer was placed in the surgeon console where both eyes were accommodated allowing greater focus and concentration, reducing fatigue during surgery. By using a new 3D endoscope, the use of two 5mm scopes inside the 12mm telescope, the image was projected onto two screens synchronized and creating a truly 3D visualization without the necessity of using specific goggles. The first da Vinci robot approved by the FDA in 2000 was composed of three arms with endoscope attachment to one of them and two instruments. Two years later, in 2002, foreseeing the necessity and value of an extra instrument in the surgical field, a four-arm robotic version was approved for clinical use. This arm would give the possibility of controlling and improving the exposure of anatomical structures and reducing dependence on a surgical assistant. At the console, two handles controlled by the surgeon were precisely connected to the arms transmitting the movements of the “master” to the robotic arms. Hand tremors were eliminated and a device that scales down movements from 1:1 to 5:1 allowed finesse according to the surgeons’ necessity. Also, the console had in its bottom a pedal unit to allow different uses of energy, such as monopolar or bipolar. 

**Figure 3:**
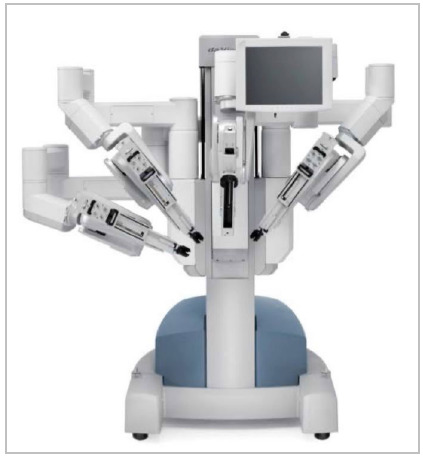
Robot da Vinci S model released in 2006.

**Figure 4:**
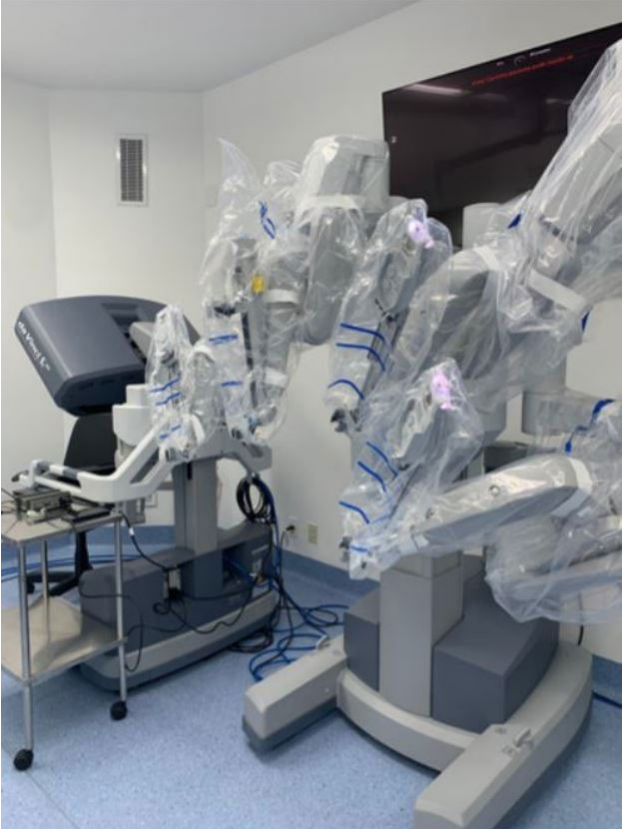
Third generation model of da Vinci: Si model.

Not completely satisfied with the first prototype, Intuitive Surgical introduced in 2006 the da Vinci S platform offering a 3D high-definition (HD) camera vision with a simplified set-up and an interactive touch screen display (Figure 3). Three years later, in 2009, the da Vinci Si model was released becoming what could be one of the most worldwide disseminated platforms since its creation (Figure 4). The new Si platform offered the concept of dual console surgery, optimizing the surgeon’s potential intraoperatively as well as introducing a reproducible and supervised manner of simulation and training for non-expert surgeons. Also, the Si robot had an upgrade of the image system thanks to the incorporation of the Tile-Pro software, and it allowed real time fluorescence imaging with the Firefly technology, playing an important role in its promulgation for minimally invasive surgery. Still, in 2011, platform adjustments and specific instruments were developed to allow a single-port access, overcoming laparoscopic limitations. 

**Figure 5:**
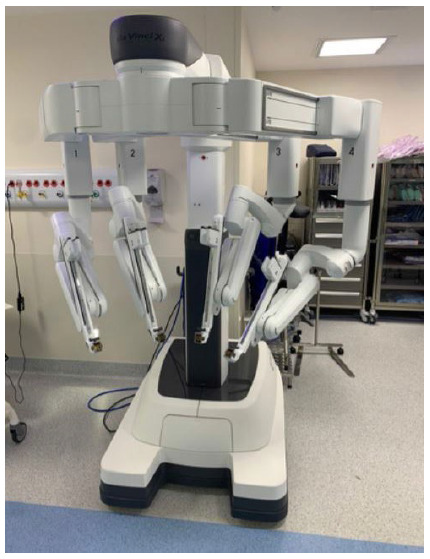
Intuitive Surgical fourth generation robot, da Vinci Xi System.

**Figure 6:**
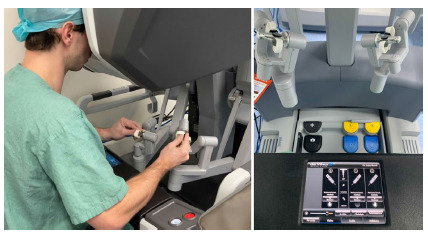
Da Vinci’s console and surgeon’s interactivity with a binocular visualization, instruments and pedals.

The most capable system created by Intuitive Surgical until is now the da Vinci Xi platform (Figures 5,6). Its release occurred in 2014, entering the market as the most advanced instrumentation, vision, cart design, as well as table motion and setup automation (Figures 5,6). Looking at a different perspective, the Xi latest model reinvented the concept of the patient cart design and its versatility and flexibility. Although impressive, the Si platform and its upgrades were not without its limitations. The robotic arms in the patients’ cart were large, making them troublesome to work and frequently leading to external collisions. Also, the robotic exoskeleton was based in a large and vertical column, having its arms parallel to each other. The 12mm robotic scope had a specific designed arm that allowed its clutching due to its different clutch model and diameter, bigger than the 8mm ports where instruments were inserted. Another limitation about the Si platform was related to the multiquadrant surgery and its docking. Whenever using different quadrants in the same procedure, the robotic arms were required to be undocked, the patient cart had to be moved and readjusted to the new desired quadrant and again redocked, increasing surgery time, prolonging anesthesia and operative room time. The most advanced multi-port da Vinci Xi model has overcome the majority of the patient cart and arm limitations in the previous prototype as well as has come up with new technology, upgrading even more the robotic surgery. 

The Intuitive Surgical fourth generation robot, da Vinci Xi System, has a brand-new designed patient cart to assure maximum mobility and flexibility during surgery. Its boom-mounted architecture provides docking from any angle and improves access around the patient at any quadrant. By targeting the surgical field and positioning the endoscope in the desired area, the robotic arms are disposed in the optimal setup. The redesigned arms provide greater internal range of motion while improving patient access and minimizing external collisions. While the earlier generations of da Vinci robots required their external arms to be widely spaced in order to maximize the working field, the opposite is true for the Xi. Its flex joints are compacted, leaving one-fist-width spacing between each arm, and it can be optimized by adjustments that can be made to the patient clearance joints of each robot arm. The new patient cart disposes of a single-fin attachment system making the docking easier and faster. Multiple ergonomic adjustments provide comfort and reduce fatigue during surgical procedures and the surgeon’s hand movements are scaled, filtered, and seamlessly translated to the instrument tips for precise instrument control.

Visualization technology represented a major step in the new platform, assuring a stable, immersive, highly magnified 3D-HD view of the surgical field while providing the surgeon autonomous, independent control of an 8-mm endoscope. Compared to the bulky scope of the earlier systems, the new designed 8-mm camera offers the surgeon a clearer view of the surgical field with a brighter image, higher resolution and longer scope. Different from the third generation, the Xi 30° endoscope can be inverted from the surgeon console without the assistant having to remove and reinstall it. Also, there is no longer a need for draping, focusing, white balancing, or calibration during surgery. By having four independent and identical robotic arms, a multiport surgery is achieved allowing instruments and endoscope to be versatile and repositioned at any time or port, if needed. Moreover, the FireFly® fluorescence imaging technology is integrated to improve specific real time decision-making steps during surgery, such as tissue perfusion or bile duct visualization. Regarding patients positioning, and patients cart mobility, the integrated table motion (ITM) feature is also a new available technology, which allows the patient to be dynamically positioned while surgery is in progress. This artifice can be performed without removing the instruments or undocking the ports, insofar in multiquadrant procedures, gravity can provide optimal exposure of the abdominal cavity. 

Another important upgrade in the new Xi system is related to its instruments, energy and stapling. First entry accessories such as robotic ports were redesigned in a fully reusable stainless steel canulae with bladeless or blunt tip, having Hasson cone and bladeless optical obturator available. Endowrist technology instruments were also enhanced, allowing robotic suction and irrigation or clip applying. Da Vinci energy devices have amplified performance, increased efficiency without sacrificing results with the speed, precision, and versatility. A second generation of bipolar instrument, the Vessel Sealer Extend, assures the surgeon to seal and cut vessels up to 7mm in diameter. Robotic stapling has been improved with complete control, full articulation, and intelligent feedback of the tissue depth, providing constant monitoring and real-time feedback. Already available in the Si model, the single-site technology has also been improved with curved internal design triangulate instruments, and its separate external arms, maximizing range of motion and minimizing collisions with the lesser bulky cart. Also, a wristed needle driver allows surgeons additional dexterity while suturing in the scenario, which was not available in the previous prototype. 

The current Robotic Era has already shown huge impact on the surgical field, and it is part of a natural and logical evolution of minimally invasive surgery. Robotic-assisted surgery is spreading quickly and has been shown to overcome the intrinsic limitations of laparoscopy. High definition, three-dimensional stereoscopic vision and magnification, a stable and surgeon guided camera, improved ergonomics, superior range of motion and motion scaling are remarkable advantages^14^. The latest data have related its benefits to visceral surgery, urology and colorectal fields^15^ ^,^ ^16^. Concerning ventral hernia repairs, encouraging outcomes have been described allowing even more complex abdominal wall reconstructions in a minimally invasive approach by the robotic platform^17^. The gynecological field has also seen benefits from robotic assisted surgery, which appears to facilitate the surgical approach for technically challenging operations to treat selected cases of cervical, endometrial, ovarian cancer or endometriosis^18^ ^,^ ^19^. Currently, most issues confronting robotic assisted procedures are especially related to its costs and longer operative time. Similar to any previous innovation or technology development, initially thought to be unaffordable, it is possible that further analysis will report to be cost-effective.

### A New Era?

The evolution and future of the robotic technology is probably based on improvement of hardware and its softwares. Robotic-assisted surgery is currently used in all surgical disciplines. Technology seeking smaller size of instruments and carts, easier and faster docking, automatic instrument exchanges; tissue-feedback technology, integration with radiological images and artificial intelligence are in perspective. For the last 20 years, the dominant used robot in robotic-assisted surgery has been Da Vinci by Intuitive Surgical. In 2020, there are more than 5,700 da Vinci units worldwide - in the United States, Europe, Asia and in the rest of the world^20^. Presumably, these developments will be pushed forward and accelerated by a very soon competitive market for manufacturers. Recently, Intuitive Surgical released its new model of robot, the da Vinci Single-Port (SP) platform. In 2018, the SP system was approved by the FDA for its use in urology patients and since then, several case reports have been described achieving successful approaches to complex urological procedures, including prostatectomy, donor nephrectomy and cystectomy^21^ ^,^ ^22^. 

Over the last years, few companies have been trying to develop robotic systems to challenge the hegemony of the Da Vinci, although not competitive yet. Titan Medical developed a Single Port Orifice Robotic Technology (SPORT) system with multi-articulated instruments, a flexible endoscope and patients cart added to a workstation in a master-slave concept. In 2018, a variety of abdominal procedures were safely completed with the SPORT prototype, in the preclinical setting showing its feasibility^23^. Another company, Transenterix, developed a robotic model called Surgibot, having its approval by the FDA denied in 2016 and in a short time, agreed to deal its assets to the Chinese MedTech investment firm Great Belief International for $29 million in 2017. Nowadays, Transenterix’s most recent technology is the Senhance platform, a digital interface between robotic and laparoscopy, with eye tracking camera control and haptic sensing in a different open-platform architecture, with three arms in different carts and an open console. Aurishealth, a part of Johnson & Johnson (J&J) Medical Devices Companies, recently introduced the Monarch™ platform, integrating the latest advancements in robotic endoscopy and bronchoscopy. A United Kingdom company, Cambridge Medical Robotics (CMR), in 2014, created and launched the Versius® robotic system, approved for its use across Europe, however not cleared for sale in the United States. (USA). The Versius technology is also based on a multiport procedure robot having not only one patient cart but four, in a more flexible concept. REVO-I, a Korean surgical platform by Meere Company South Korea entered the market in 2017 after the Korean FDA approval in a very similar da Vinci Si prototype, and seeking a more cost-effective model. It consists of a surgeon control console, a four-armed robotic operation cart, an HD vision cart and reusable endoscopic instruments. Five years ago, Verb Surgical was founded after Google and J&J announced a joint venture for development of their surgical robotic system, still in progress. 

## CONCLUSION

Robotic surgery is a rapidly evolving technology and has a very entertaining history. Intuitive Surgical stands as its major protagonist, but despite its hegemony and dominance, the evolution of robotic surgery is far from over, with multiple potential competitors on the horizon expanding the boundaries of the optimal technology. In this review, we aimed to describe the evolution of the robotic surgical systems, in the last years. Surgeons’ higher interface with robots will not only be achieved with new tools, but by artificial intelligence integration and a new way to interpret modern surgery, a paradigm shift well worth to be followed.

## References

[1] Zajtchuk R, Rellamy RF, Grande CM (1995). Anesthesia and perioperative care of the combat casualty. Part IV - surgical combat casualty care. Textbook of Military Medicine..

[2] Kwoh YS, Hou J, Jonckheere EA, Hayati S (1988). A robot with improved absolute positioning accuracy for CT guided stereotactic brain surgery. IEEE Trans Biomed Eng.

[3] Davies BL, Hibberd RD, Ng WS, Timoney AG, Wickham JE (1991). The development of a surgeon robot for prostatectomies. Proc Inst Mech Eng H.

[4] Paul HA, Bargar WL, Mittlestadt B, Musits B, Taylor RH, Kazanzides P (1992). Development of a surgical robot for cementless total hip arthroplasty. Clin Orthop Relat Res.

[5] Kavoussi LR, Moore RG, Adams JB, Partin AW (1995). Comparison of robotic versus human laparoscopic camera control. J Urol.

[6] Falcone T, Goldberg J, Garcia-Ruiz A, Margossian H, Stevens L (1999). Full robotic assistance for laparoscopic tubal anastomosis a case report. J Laparoendosc Adv Surg Tech A.

[7] Marescaux J, Leroy J, Gagner M, Rubino F, Mutter D, Vix M (2001). Transatlantic robot-assisted telesurgery. Nature.

[8] DiMaio S, Hanuschik M, Kreaden U, Rosen J, Hannaford B, Satava RM (2011). Surgical Robotics: Systems Applications and Visions..

[9] Himpens J, Leman G, Cadiere G (1998). Telesurgical laparoscopic cholecystectomy. Surg Endosc.

[10] Himpens J (2012). Surgery in space: the future of robotic telesurgery (Haidegger T, Szandor J, Benyo Z. Surg Endosc. 2011;25(3):681-690). Surg Endosc.

[11] Cadiere GB, Himpens J, Vertruyen M, Favretti F (1999). The world's first obesity surgery performed by a surgeon at a distance. Obes Surg.

[12] Salisbury JK (1998). The heart of microsurgery. Mech Eng.

[13] Parekattil SJ, Moran ME (2010). Robotic instrumentation evolution and microsurgical applications. Indian J Urol.

[14] Damle A, Damle RN, Flahive JM, Schlussel AT, Davids JS, Sturrock PR (2017). Diffusion of technology Trends in robotic-assisted colorectal surgery. Am J Surg.

[15] Bonet X, Ogaya-Pinies G, Woodlief T, Hernandez-Cardona E, Ganapathi H, Rogers T (2018). Nerve-sparing in salvage robot-assisted prostatectomy surgical technique, oncological and functional outcomes at a single high-volume institution. BJU Int.

[16] Zhu XL, Yan PJ, Yao L, Liu R, Wu DW, Du BB (2019). Comparison of short-term outcomes between robotic-assisted and laparoscopic surgery in colorectal cancer. Surg Innov.

[17] Morrell ALG, Morrell AC, Cavazzola LT, Pereira GSS, Mendes JM, Abdalla RZ (2020). Robotic assisted eTEP ventral hernia repair: Brazilian early experience. Hernia.

[18] Yim GW, Kim YT (2012). Robotic surgery in gynecologic cancer. Curr Opin Obstet Gynecol.

[19] Morrell ALG, Ribeiro GMPAR, Santos TP, Morrell AC, Chamie LP, Frare N (2020). Robotic Natural Orifice Specimen Extraction with Totally Intracorporeal Anastomosis Associated with Firefly Fluorescence Bowel Resection for Deep Infiltrating Endometriosis. J Gynecol Surg.

[20] Intuitive.com https://isrg.intuitive.com.

[21] LaMattina JC, Alvarez-Casas J, Lu I, Powell JM, Sultan S, Phelan MW (2018). Robotic-assisted single-port donor nephrectomy using the da Vinci single-site platform. J Surg Res.

[22] Gaboardi F, Pini G, Suardi N (2019). Robotic laparoendoscopic single-site radical prostatectomy (R-LESS-RP) with daVinci Single-Site(r) platform Concept and evolution of the technique following an IDEAL phase 1. J Robot Surg.

[23] Seeliger B, Diana M, Ruurda JP, Konstantinidis KM, Marescaux J, Swanström LL (2019). Enabling single-site laparoscopy the SPORT platform. Surg Endosc.

